# Relationships Between Cardinal Features of Obstructive Sleep Apnea and Blood Pressure: A Retrospective Study

**DOI:** 10.3389/fpsyt.2022.846275

**Published:** 2022-04-08

**Authors:** Yunyan Xia, Kai You, Yuanping Xiong

**Affiliations:** ^1^Department of Otorhinolaryngology–Head and Neck Surgery, First Affiliated Hospital of Nanchang University, Nanchang, China; ^2^Department of Anesthesiology, First Affiliated Hospital of Nanchang University, Nanchang, China

**Keywords:** obstructive sleep apnea, blood pressure, intermittent hypoxia, sleep fragmentation, interaction

## Abstract

**Background:**

Obstructive sleep apnea (OSA) is associated with hypertension; however, the associations between cardinal features of OSA, such as intermittent hypoxia (IH) and sleep fragmentation (SF), and blood pressure remain unclear. We performed this study to address this issue.

**Method:**

We investigated 335 subjects with the polysomnography (PSG) tests. Data, including basic characteristics, PSG parameters, and blood pressure, were collected. We calculated *p*-values for linear trends of blood pressure across oxygen-desaturation index (ODI)/microarousal index (MAI) quartiles. Logistic regressions were used to determine the risk factors for abnormal blood pressure and to detect the multiplicative interaction between ODI and MAI with blood pressure.

**Results:**

After adjusting for multiple variables, compared with subjects with lower ODI quartiles, those with higher ODI quartiles had significant higher systolic blood pressure (SBP) and diastolic blood pressure (DBP) (p for trend = 0.010 and 0.018, respectively). And compared with subjects with lower ODI quartiles, those with higher ODI quartiles were also more likely to have abnormal DBP and hypertension after adjusting for multiple variables. Similarly, compared with subjects with lower MAI quartiles, those with higher MAI quartiles had significant higher SBP and DBP, and were more likely to have abnormal DBP and hypertension. No significant multiplicative interactions between ODI and MAI with blood pressure were detected.

**Conclusion:**

Subjects with more severe IH/SF had significant higher blood pressure and were more likely to have abnormal DBP and hypertension than those with less severe IH/SF. No interaction between IH and SF on the relationship with blood pressure was shown.

## Introduction

Obstructive sleep apnea (OSA) is a common sleep disorder and its prevalence increases with age and obesity (1). A recent study reported a 23.4 and 49.7% prevalence of moderate-to-severe OSA in females and males, respectively (2). Multiple studies have demonstrated an association between OSA and cardiovascular diseases, particularly hypertension. The Sleep Heart Health study reported a prevalence of 59, 62, and 67% for hypertension in mild, moderate, and severe OSA, respectively (3). Meanwhile, OSA is particularly common in refractory hypertension (4, 5). Studies have also demonstrated an independent relationship between OSA severity and blood pressure. A large cross-sectional study demonstrated that, after adjusting for age, body mass index (BMI), and gender, OSA severity index was a significant predictor of both systolic (SBP) and diastolic (DBP) blood pressures, and each additional apneic event per hour of sleep increased the odds of hypertension by about 1% (6). A linear increase in SBP and DBP with increasing OSA severity was also demonstrated after adjusting for age, BMI, and gender (7).

Obstructive sleep apnea is characterized by two cardinal pathophysiological features, including intermittent hypoxia (IH) and sleep fragmentation (SF). IH has long been recognized as one of the mechanisms in the development of OSA-related hypertension, and rodent studies have demonstrated a robust association between IH and hypertension (8). It has been suggested that IH may increase the sympathetic nervous system and renin-angiotensin system activity, (9–11) cause endothelial dysfunction, (12, 13) and contribute to inflammation and metabolic disorders (14, 15) to induce hypertension. Recent evidence also implicates SF as a distinct trigger for elevated blood pressure. Rodent studies have demonstrated that SF contributes to sympathetic activation and endothelial dysfunction, which promotes the development of hypertension (16). However, the results of clinical studies have been inconsistent. Some studies have demonstrated a positive association between SF and the risk for hypertension, (17–19) while others have found no effects of SF on blood pressure (20–22). Most of these clinical studies had some limitations, including small sample sizes (19–21) or focusing on specific populations (17, 18). And whether the risk of hypertension increases with the elevating severity of IH/SF is not yet known. In addition, the interaction between IH and SF in the association with blood pressure has not been evaluated.

We performed this study to address these gaps. We aimed to deeply assess the independent associations of the two cardinal features of OSA, IH and SF, with blood pressure, and the effects of the interaction between IH and SF.

## Materials and Methods

### Subjects

**FIGURE 1:**
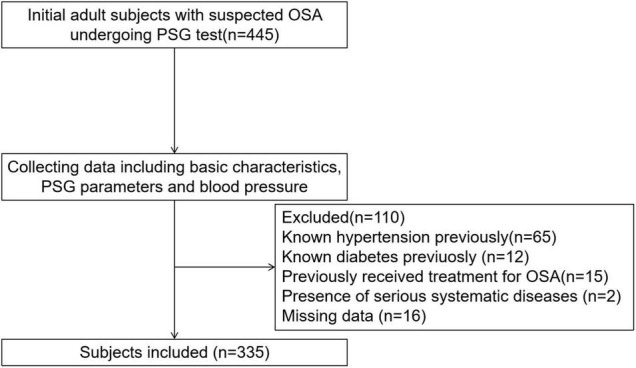
Enrollment flow chart for the study population. OSA, obstructive sleep apnea; PSG, polysomnography.

Adult inpatients with suspected OSA who underwent polysomnography (PSG) between January 2014 and January 2021 at the First Affiliated Hospital of Nanchang University were included. The following criteria were used to exclude 110 subjects: known hypertension previously (*n* = 65); known diabetes mellitus previously (*n* = 12); previous OSA treatment (*n* = 15); serious systemic diseases (e.g., heart failure or renal failure) (*n* = 2); and missing data (*n* = 16). A total of 335 patients with complete data were included (Figure 1).

### Basic Characteristics

Comprehensive medical histories and blood tests were obtained from the subjects. The medical history included gender, age, hypertension, diabetes, other diseases, and smoking and alcohol consumption. Weights and heights were recorded and BMI was calculated as weight (kg)/height (m^2^). Blood tests were also performed, including fasting glucose levels.

### Overnight Polysomnography Parameters

The subjects received a standard laboratory-based PSG (Alice 4 or 5; Respironics, Pittsburgh, PA, United States). Electroencephalogram (EEG), electrooculogram (EOG), electrocardiogram (ECG), electromyogram (EMG), nasal and oral airflow, thoracic and abdominal respiratory effort, pulse oximetry, posture, and snoring data were obtained. Sleep stages, respiratory events, and microarousals were scored automatically using a computer software and were subsequently checked manually by a skilled technician, following the American Academic Sleep Medicine (AASM) criteria. The apnea–hypopnea index (AHI) was defined as the number of apnea and hypopnea events per hour during sleep. The oxygen-desaturation index (ODI) was defined as the number of times per hour of sleep that the blood oxygen level dropped by ≥ 4% from the baseline. The microarousal index (MAI) was defined as the number of arousals per hour of sleep, and an arousal was defined as an abrupt shift in the EEG frequency, including alpha, theta and/or frequencies > 16Hz (but not spindles) that lasted at least 3 s, with at least 10 s of stable sleep preceding the change. The lowest oxygen saturation value during sleep was referred to as the LSpO_2_ (23). As AHI and ODI have strong collinearity and both of them can represent IH, we chose one of them (ODI) to perform the analyses. We chose MAI to represent SF.

### Blood Pressure

Three daytime blood pressure measurements were recorded after at least 5 min of rest in a sitting position in accordance with the American Society of Hypertension guidelines using a mercury sphygmomanometer, and their mean was calculated. The blood pressure threshold for hypertension was 140/90 mmHg. According to the Guidelines for Prevention and Treatment of Hypertension in China, hypertension was defined as abnormal SBP (≥ 140 mmHg) or DBP (≥ 90 mmHg) or both (24).

### Statistical Analysis

Continuous data with a normal distribution were presented as means ± standard deviation, while those with skewed distribution were presented as medians (with interquartile range). Categorical data were presented as numbers (%). The *p*-values for trends of basic characteristic, PSG parameters and blood pressure across ODI quartiles or MAI quartiles were calculated using the polynomial linear trend test for continuous variables and the linear-by-linear association test for dichotomous variables. Binary logistic regression analyses were used to determine the risk factors for abnormal SBP, abnormal DBP, and hypertension, along with the linear trends of the risk of abnormal blood pressure across ODI quartiles or MAI quartiles. Multiplicative interactions were detected with logistic regression. All statistical analyses were performed using SPSS Statistics 21.0 software (IBM Corp., Armonk, NY, United States). *P* < 0.05 was taken to indicate statistical significance.

## Results

### Basic Characteristics

The 335 subjects included in this study were categorized in the basis of ODI (≤ 9.3, 9.3–33.7, 33.7–66.4, and > 66.4) and MAI (≤ 10.3, 10.3–18.7, 18.7–36.6, and > 36.6) quartiles. Most subjects with a high ODI were obese males, and this group had a higher percentage of drinkers, higher glucose levels, and more severe OSA (demonstrated by higher AHI, ODI, and MAI levels, and lower LSpO_2_ levels) (all *p*-values < 0.05) (Table 1). The percentages of subjects with abnormal SBP, abnormal DBP, and hypertension increased from 7.1 to 39.8%, from 1.2 to 50.6%, and from 7.1 to 60.2%, respectively, across the ODI quartiles (all *p*-values < 0.001) (Table 1). Similarly, most of the subjects with a high MAI were obese males, and this group had higher glucose levels and more severe OSA (all *p*-values < 0.05) (Table 2). The percentages of subjects with abnormal SBP, abnormal DBP, and hypertension increased from 9.5 to 43.4%, from 2.4 to 53.0%, and from 10.7 to 61.4%, respectively, across MAI quartiles (all *p*-values < 0.001) (Table 2).

**TABLE 1:** Characteristics and blood pressure of included subjects according to ODI quartiles.

	Whole population (*N* = 335)	ODI ≤ 9.3 (*n* = 84)	9.3 < ODI ≤ 33.7 (*N* = 84)	33.7 < ODI ≤ 66.4 (*N* = 84)	ODI > 66.4 (*N* = 83)	*p* for trend
**Basic characteristics**						
Male, N (%)	285 (85.1)	64 (76.2)	71 (84.5)	79 (94.0)	71 (85.5)	0.031
Age, y	43.0(33.0,51.0)	41.0(31.3,50.0)	49.0(37.3,55.0)	41.0(33.0,50.0)	40.0(32.0,47.0)	0.254
BMI, Kg/m^2^	24.5(26.4,28.7)	26.0(23.2,27.3)	26.0(24.1,27.9)	27.0(24.2,28.7)	27.8(25.5,30.1)	<0.001
Smoking, N (%)	110 (32.8)	27 (32.1)	30 (35.7)	28 (33.3)	25 (30.1)	0.715
Drinking, N (%)	57 (17.0)	21 (25.0)	13 (15.5)	12 (14.3)	11 (13.3)	0.047
Glucose	5.15(4.70,5.68)	5.12(4.69,5.41)	4.95(4.71,5.48)	5.11(4.57,5.70)	5.54(4.98,6.05)	0.001
**PSG parameters**						
AHI	32.9(9.3,64.9)	3.4(1.4,6.2)	18.9(12.4,25.6)	49.8(40.0,59.0)	75.0(71.2,87.5)	<0.001
ODI	33.7(9.3,66.4)	3.6(1.3,5.9)	20.4(13.1,26.3)	52.7(40.2,60.7)	81.5(73.1,87.5)	<0.001
MAI	18.7(10.3,36.6)	7.0(3.4,12.3)	14.9(9.5,19.5)	25.9(17.9,36.2)	52.5(31.6,65.9)	<0.001
LSpO_2_	80.0(67.0,87.0)	90.0(87.0,93.0)	84.0(77.3,87.0)	74.0(61.0,81.0)	62.0(53.0,71.3)	<0.001
**Blood pressure**						
SBP, mmHg	126.0(115.0,138.0)	116.5(108.3,126.0)	123.0(111.5,134.8)	130.0(118.0,139.0)	136.0(124.0,145.0)	0.010
DBP, mmHg	80.0(72.0,89.0)	75.5(69.3,82.0)	76.0(69.0,85.0)	81.5(74.3,90.0)	90.0(80.0,95.0)	0.018
Abnormal SBP, N(%)	75 (22.1)	6 (7.1)	15 (17.9)	20 (23.8)	33 (39.8)	<0.001
Abnormal DBP, N(%)	76 (22.7)	1 (1.2)	12 (14.3)	21 (25.0)	42 (50.6)	<0.001
Hypertension, N(%)	101 (30.1)	6 (7.1)	17 (20.2)	28 (33.3)	50 (60.2)	<0.001

*p for trend was tested using the polynomial linear trend test for continuous variables, and by the linear-by linear association test for dichotomous variables. p for trend for SBP and DBP were adjusted for gender, age, BMI, smoking status, drinking status, glucose level and MAI. ODI, oxygen-desaturation index; BMI, body mass index; PSG, polysomnography; AHI, apnea-hypopnea index; MAI, microarousal index; LSpO_2_, lowest whole oxygen saturation value during sleep; SBP, systolic blood pressure; DBP, diastolic blood pressure.*

**TABLE 2:** Characteristics and blood pressure of included subjects according to MAI quartiles.

	MAI ≤ 10.3 (*n* = 84)	10.3 < MAI ≤ 18.7 (*n* = 84)	18.7 < MAI ≤ 36.6 (*n* = 84)	MAI > 36.6 (*n* = 83)	*p* for trend
**Basic characteristics**					
Male, N (%)	62 (26.2)	74 (88.1)	74 (88.1)	75 (90.4)	0.004
Age, y	41.5 (30.3, 51.8)	44.0 (34.3, 51.0)	32.5 (43.5, 51.8)	41.0 (35.0, 50.1)	0.901
BMI, Kg/m^2^	25.8 (23.4, 27.3)	26.5 (24.5, 28.7)	26.7 (24.2, 28.4)	27.6 (25.7, 30.1)	<0.001
Smoking, N(%)	26 (31.0)	28 (33.3)	27 (32.1)	29 (34.9)	0.640
Drinking, N(%)	15 (17.9)	18 (21.4)	12 (14.3)	12 (14.5)	0.346
Glucose	4.98 (4.48, 5.41)	5.10 (4.76, 5.49)	5.13 (4.76, 5.67)	5.55 (4.87, 6.05)	0.003
**PSG parameters**					
AHI	4.8 (1.6, 10.8)	21.3 (9.8, 40.3)	48.1 (24.5, 60.4)	73.3 (64.3, 81.1)	<0.001
ODI	5.5 (1.4, 10.9)	23.1 (10.4, 41.3)	46.5 (26.3, 64.9)	75.3 (65.4, 84.7)	<0.001
MAI	5.7 (2.2, 8.4)	15.1 (12.9, 16.9)	25.8 (22.6, 30.4)	54.1 (45.1, 70.0)	<0.001
LSpO_2_	89.0 (85.0, 91.8)	82.0 (75.3, 87.0)	74.5 (61.8, 82.8)	64.0 (54.0, 74.0)	<0.001
**Blood pressure**					
SBP, mmHg	119.0 (108.3, 127.8)	125.0 (116.0, 136.0)	125.0 (115.0, 138.0)	137.0 (126.0, 147.0)	0.008
DBP, mmHg	75.0 (69.3, 82.0)	79.5 (73.0, 87.0)	80.0 (71.0, 88.8)	90.0 (80.0, 97.0)	0.011
Abnormal SBP, N (%)	8 (9.5)	11 (13.1)	19 (22.6)	36 (43.4)	<0.001
Abnormal DBP, N (%)	2 (2.4)	13 (15.5)	17 (20.2)	44 (53.0)	<0.001
Hypertension, N (%)	9 (10.7)	16 (19.0)	25 (29.8)	51 (61.4)	<0.001

*p for trend was tested using the polynomial linear trend test for continuous variables, and by the linear-by linear association test for dichotomous variables. p for trend for SBP and DBP were adjusted for gender, age, BMI, smoking status, drinking status, glucose level and ODI. MAI, microarousal index; BMI, body mass index; PSG, polysomnography; AHI, apnea-hypopnea index; ODI, oxygen-desaturation index; LSpO_2_, lowest whole oxygen saturation value during sleep; SBP, systolic blood pressure; DBP, diastolic blood pressure.*

### Oxygen-Desaturation Index and Blood Pressure

**FIGURE 2:**
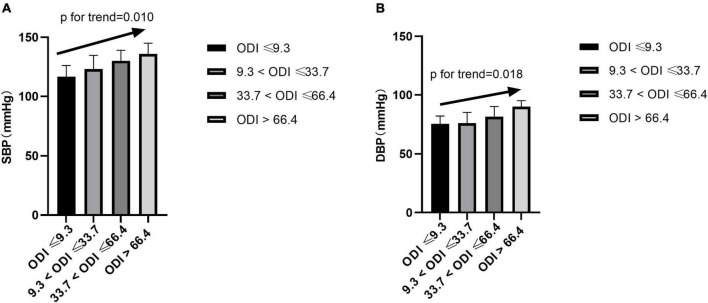
Blood pressure across ODI quartiles. SBP, systolic blood pressure; DBP, diastolic blood pressure; ODI, oxygen-desaturation index.

After adjusting for gender, age, BMI, smoking status, drinking status, glucose levels, and MAI, compared with subjects with lower ODI quartiles, those with higher ODI quartiles had significant higher SBP and DBP (*p* for trend = 0.010 and 0.018, respectively) (Table 1 and Figures 2A,B). Logistic regression analyses showed that compared with subjects with lower ODI quartiles, those with higher ODI quartiles were more likely to have abnormal DBP, and a significant linear trend for the risk of abnormal DBP and increasing ODI quartiles was seen (Table 3 and Model 1), with odds ratios (ORs) [95% confidential interval (CI)] of 1 (reference), 9.893 (1.233, 79.394), 11.985 (1.490, 96.425), and 20.387 (2.354, 176.545), respectively (*p* for trend = 0.047). Although no significant linear trend for hypertension with increasing ODI quartiles was seen (*p* for trend = 0.087), compared with subjects with the lowest ODI quartile, those with higher ODI quartiles were more likely to have hypertension [33.7 < ODI ≤ 66.4 vs. ODI ≤ 9.3, OR (95%CI) = 3.016 (1.046, 8.696), *p* = 0.041; and ODI > 66.4 vs. ODI ≤ 9.3, OR (95%CI) = 4.759 (1.436, 15.770), *p* = 0.011] (Table 3 and Model 1).

**TABLE 3:** Logistic regression models of selected factors and abnormal blood pressure.

	Abnormal SBP		Abnormal DBP		Hypertension	
	OR (95%CI)	p	OR (95%CI)	p	OR (95%CI)	p
**Model 1**						
ODI ≤ 9.3	1		1		1	
9.3 < ODI ≤ 33.7	1.947 (0.684, 5.538)	0.212	9.893 (1.233, 79.394)	0.031	2.253 (0.805, 6.306)	0.122
33.7 < ODI ≤ 66.4	2.055 (0.688, 6.136)	0.197	11.985 (1.490, 96.425)	0.020	3.016 (1.046, 8.696)	0.041
ODI > 66.4	2.563 (0.740, 8.878)	0.138	20.387 (2.354, 176.545)	0.006	4.759 (1.436, 15.770)	0.011
*p* for trend	0.497		0.047		0.087	
**Model 2**						
MAI ≤ 10.3	1		1		1	
10.3 < MAI ≤ 18.7	1.104 (0.382, 3.194)	0.855	5.388 (1.113, 26.088)	0.036	1.157 (0.434, 3.086)	0.770
18.7 < MAI ≤ 36.6	1.766 (0.591, 5.280)	0.308	6.342 (1.268, 31.717)	0.025	1.555 (0.558, 4.335)	0.398
MAI > 36.6	3.121 (0.880, 11.063)	0.078	19.137 (3.474, 105.427)	0.001	3.347 (1.033, 10.844)	0.044
*p* for trend	0.193		0.002		0.082	

*SBP, systolic blood pressure; DBP, diastolic blood pressure; ODI, oxygen-desaturation index; MAI, microarousal index; OR, odd ratio; CI, confidential interval.*

### Microarousal Index and Blood Pressure

**FIGURE 3:**
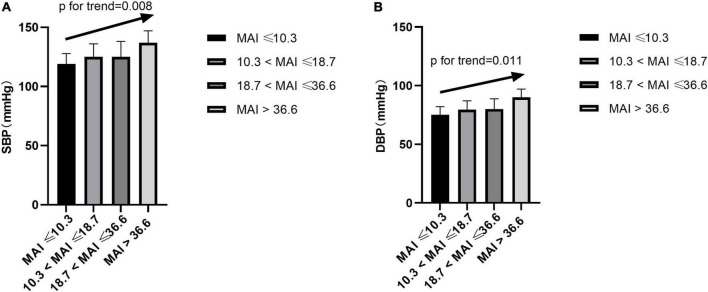
Blood pressure across MAI quartiles. SBP, systolic blood pressure; DBP, diastolic blood pressure; MAI, microarousal index.

After adjusting for multiple variables, including ODI, compared with subjects with lower MAI quartiles, those with higher MAI quartiles had significant higher SBP and DBP (*p* = for trend 0.008 and 0.011, respectively) (Table 2 and Figures 3A,B). Logistic regression analyses showed that compared with subjects with lower MAI quartiles, those with higher MAI quartiles were more likely to have abnormal DBP, and a significant linear trend for the risk of abnormal DBP and increasing MAI quartiles was seen (Table 3 and Model 2), with ORs (95% CI) of 1 (reference), 5.388 (1.113, 26.088), 6.342 (1.268, 31.717), and 19.137 (3.474, 105.427) (*p* for trend = 0.002). Although no significant linear trend for hypertension with increasing MAI quartiles was seen (*p* for trend = 0.082), compared with subjects with the lowest MAI quartile, those with the highest MAI quartile were more likely to have hypertension [MAI > 36.6 vs. MAI ≤ 10.3, OR (95%CI) = 3.347 (1.033, 10.844), *p* = 0.044] (Table 3 and Model 2).

### Oxygen-Desaturation Index and Microarousal Index Interaction

We investigated the effect of a multiplicative interaction between MAI and ODI on blood pressure. After adjusting for multiple variables, there were no statistically significant interactions, with ORs (95%CI) of 1.121 (0.832, 1.510), 0.768 (0.523, 1.129), and 1.098 (0.825, 1.463) for abnormal SBP, abnormal DBP, and hypertension, respectively (Table 4).

**TABLE 4:** Multiplicative interaction between ODI and MAI.

	Adjusted OR (95%CI)	*p*
Abnormal SBP	1.121 (0.832, 1.510)	0.453
Abnormal DBP	0.768 (0.523, 1.129)	0.179
Hypertension	1.098 (0.825, 1.463)	0.521

*The gender, age, BMI, smoking status, drinking status, glucose level, ODI quartiles, MAI quartiles and ODI × MAI were added into the model. SBP, systolic blood pressure; DBP, diastolic blood pressure; ODI, oxygen-desaturation index; MAI, microarousal index; OR, odd ratio; CI, confidential interval.*

## Discussion

We found that after adjusting for multiple variables, compared with subjects with lower ODI quartiles, those with higher ODI quartiles had significant higher SBP and DBP. Logistic regression analyses showed that compared with subjects with lower ODI quartiles, those with higher ODI quartiles were more likely to have abnormal DBP and hypertension after adjusting for multiple variables. Similarly, compared with subjects with lower MAI quartiles, those with higher MAI quartiles had significant higher SBP and DBP, and were more likely to have abnormal DBP and hypertension. Furthermore, no significant multiplicative interaction of ODI and MAI on blood pressure was detected.

In this study, we found that those with more severe IH (represented by ODI) had significant higher SBP and DBP, and were more likely to have abnormal DBP and hypertension than those with less severe IH. An increasing number of studies have demonstrated that IH can induce hypertension. In human experimental models, hypoxia has been shown to lead to measurable elevations in blood pressure and sympathetic activation (25–28). IH can stimulate the chemoreceptors and carotid body, and attenuate the activation of carotid baroreceptor, resulting in increased sympathetic activation (29, 30). Persistent sympathetic activation not only elevates blood pressure by increasing vascular resistance and vascular remodeling but also causes sodium retention, impaired natriuresis, and blood pressure elevation by activating the renin-angiotensin system. Meanwhile, IH can promote endothelial dysfunction, systematic inflammation, and metabolic dysfunction, including dyslipidemia and glucose metabolism disorder, which contributes to the development of hypertension (31).

Results from previous clinical studies about the relationship between SF and blood pressure have been controversial. The PROOF-SYNAPSE study of 780 healthy older subjects found that repetitive sympathetic arousals during sleep were associated with elevated SBP and a higher risk of hypertension (17). (18) found that arousal index was an independent risk factor for DBP in male patients (18). (19) demonstrated that arousal index independently predicted a small percentage of the variance in nocturnal reduction in DBP (19). However, other studies have failed to demonstrate an independent relationship between SF and blood pressure. A study of 1021 subjects showed no independent association between SF and awake blood pressure after controlling for the influence of AHI in subjects with AHI ≥ 1. Two other studies also demonstrated that SF was not independently associated with blood pressure (20, 21). In our study, we found that those with more severe SF (represented by MAI) had significant higher SBP and DBP, and were more likely to have abnormal DBP and hypertension than those with less severe SF. A rodent study has demonstrated that long-term SF induces vascular endothelial dysfunction and mild blood pressure increase, (16) and leads to morphologic vessel changes characterized by elastic fiber disruption and disorganization, increased recruitment of inflammatory cells, and altered expression of senescence markers. This suggests a role for SF in the cardiovascular morbidity related to OSA, including hypertension (16).

In addition to the independent relationships between IH/SF and blood pressure, we also explored the multiplicative effects of IH and SF on blood pressure, and found no evidence of an interaction. This suggests that these two factors contribute independently to abnormal blood pressure.

Meanwhile, we explored the relationships between BMI and hypertension. We found those with higher BMI quartiles were more likely to have abnormal SBP and hypertension than those with lower BMI quartiles, which indicated the important role of obesity in hypertension (Supplementary Table 1). We also detected the interactions between BMI and ODI on abnormal blood pressure, and found no significant interactions (Supplementary Table 2). Furthermore, we divided the subjects into two categories according to the median BMI, and found that only in BMI > 26.37 category, those with higher MAI quartiles were more likely to have abnormal DBP, which needed to be further studied in future (Supplementary Table 3).

This study had some limitations. First, it was a retrospective study, so causality cannot be inferred. Second, the statistical power was relatively low due to the small sample size. Third, although we adjusted for several common confounding factors, other more complex factors, such as lifestyle and exercise, were not taken into consideration. Despite these limitations, the sleep data based on standard PSG and objective measurements increased the credibility of our results.

In conclusion, we found that subjects with more severe IH or SF had significant higher SBP and DBP, and were more likely to have abnormal DBP and hypertension than those with less severe IH or SF. There was no multiplicative interaction between IH and SF regarding the effects on blood pressure. Therefore, it is important to consider the roles of both SF and IH while treating hypertensive patients with OSA.

## Data Availability Statement

The raw data supporting the conclusions of this article will be made available by the authors, without undue reservation.

## Ethics Statement

This study was approved by the Internal Review Board of the Institutional Ethics Committee of the First Affiliated Hospital of Nanchang University (Approval No. 2020-12-139), and the study was conducted in accordance with all relevant tenets of the Declaration of Helsinki. This was a retrospective study. The ethics committee waived the requirement of written informed consent for participation.

## Author Contributions

YYX and YPX designed the study and wrote and manuscript. KY and YYX collected the data. KY performed the analysis. All authors contributed to the article and approved the submitted version.

## Conflict of Interest

The authors declare that the research was conducted in the absence of any commercial or financial relationships that could be construed as a potential conflict of interest.

## Publisher’s Note

All claims expressed in this article are solely those of the authors and do not necessarily represent those of their affiliated organizations, or those of the publisher, the editors and the reviewers. Any product that may be evaluated in this article, or claim that may be made by its manufacturer, is not guaranteed or endorsed by the publisher.
