# Supplementary material for: Relationships Between Cardinal Features of Obstructive Sleep Apnea and Blood Pressure: A Retrospective Study
Source: Front Psychiatry. 2022 Apr 8;13:846275. doi: 10.3389/fpsyt.2022.846275 (PMC9027567; doi:10.3389/fpsyt.2022.846275)
Supplement: Supplementary file 1 [file Table_1.DOC]

**Supplementary Table 1 Logistic regression models of selected factors and abnormal blood pressure.**

|  | Abnormal SBP | | Abnormal DBP | | Hypertension | |
| --- | --- | --- | --- | --- | --- | --- |
|  | OR(95%CI) | p | OR(95%CI) | p | OR(95%CI) | p |
| BMI ≤ 24.49 | 1 | | 1 | | 1 | |
| 24.49 <BMI≤ 26.37 | 1.260(0.511, 3.109) | 0.615 | 1.443(0.548, 3.801) | 0.458 | 1.511(0.654, 3.490) | 0.334 |
| 26.37 <BMI≤ 28.69 | 1.255(0.510, 3.087) | 0.621 | 1.615(0.767, 3.403) | 0.207 | 1.139(0.574, 2.260) | 0.710 |
| BMI> 28.69 | 2.940(1.223, 7.072) | 0.016 | 1.535(0.782, 3.010) | 0.213 | 2.217(1.178, 4.174) | 0.014 |
| p for trend | 0.044 | | 0.387 | | 0.084 | |

To observe the risks for abnormal blood pressure across BMI quartiles, the gender, age, smoking status, drinking status, glucose level, ODI and MAI were adjusted in the model.

Abbreviations: BMI, body mass index; SBP, systolic blood pressure; DBP, diastolic blood pressure; ODI, oxygen-desaturation index; MAI, microarousal index; OR, odd ratio; CI, confidential interval.

**Supplementary Table 2 Multiplicative interaction between ODI and BMI.**

|  | Adjusted OR(95%CI) | p |
| --- | --- | --- |
| Abnormal SBP | 0.835(0.639, 1.091) | 0.186 |
| Abnormal DBP | 0.781(0.569, 1.072) | 0.126 |
| Hypertension | 0.829(0.635, 1.082) | 0.168 |

The gender, age, smoking status, drinking status, glucose level, MAI, ODI quartiles, BMI quartiles and ODI*BMI were added into the model.

Abbreviations: BMI, body mass index; SBP, systolic blood pressure; DBP, diastolic blood pressure; ODI, oxygen-desaturation index; MAI, microarousal index; OR, odd ratio; CI, confidential interval.

**Supplementary Table 3 Logistic regression models of selected factors and abnormal diastolic blood pressure stratified by BMI category.**

|  | Abnormal DBP | |
| --- | --- | --- |
|  | OR(95%CI) | p |
| **Model 1** | | |
| ODI ≤ 5.75 | 1 | |
| 5.75 < ODI ≤ 21.50 | 0.000(0.000, -) | 0.998 |
| 21.50 < ODI ≤ 51.90 | 6.322(0.662, 60.420) | 0.109 |
| ODI> 51.90 | 9.963(0.911, 108.898) | 0.060 |
| p for trend | 0.313 | |
| **Model 2** | | |
| MAI ≤ 7.95 | 1 | |
| 7.95 < MAI ≤ 15.80 | 0.990(0.079, 12.355) | 0.994 |
| 15.80 < MAI ≤ 32.90 | 1.631(0.140, 18.995) | 0.696 |
| MAI > 32.90 | 1.680(0.116, 24.255) | 0.703 |
| p for trend | 0.949 | |
| **Model 3** | | |
| ODI ≤ 16.85 | 1 | |
| 16.85 < ODI ≤ 52.95 | 1.339(0.348, 5.158) | 0.671 |
| 52.95 < ODI ≤ 75.55 | 1.793(0.438, 7.344) | 0.417 |
| ODI > 75.55 | 1.068(0.204, 5.585) | 0.938 |
| p for trend | 0.726 | |
| **Model 4** | | |
| MAI ≤ 14.85 | 1 | |
| 14.85 < MAI ≤ 22.45 | 3.266(0.872, 12.224) | 0.079 |
| 22.45 < MAI ≤ 47.4 | 2.339(0.549, 9.953) | 0.250 |
| MAI > 47.4 | 10.036(1.988, 50.665) | 0.005 |
| p for trend | 0.017 | |

Model 1: To observe the risks for abnormal blood pressure across ODI quartiles in BMI ≤ 26.37 categry, the gender, age, BMI, smoking status, drinking status, glucose level and MAI were adjusted in model 1.

Model 2: To observe the risks for abnormal blood pressure across MAI quartiles BMI ≤ 26.37, the gender, age, BMI, smoking status, drinking status, glucose level and ODI were adjusted in model 2.

Model 3: To observe the risks for abnormal blood pressure across ODI quartiles in BMI > 26.37 categry, the gender, age, BMI, smoking status, drinking status, glucose level and MAI were adjusted in model 3.

Model 4: To observe the risks for abnormal blood pressure across MAI quartiles BMI > 26.37, the gender, age, BMI, smoking status, drinking status, glucose level and ODI were adjusted in model 4.

Abbreviations: BMI, body mass index; DBP, diastolic blood pressure; ODI, oxygen-desaturation index; MAI, microarousal index; OR, odd ratio; CI, confidential interval.
